# Application of fluorescence spectroscopy using classical right angle technique in white wines classification

**DOI:** 10.1038/s41598-019-54697-8

**Published:** 2019-12-03

**Authors:** Ramona-Crina Suciu, Liviu Zarbo, Francois Guyon, Dana Alina Magdas

**Affiliations:** 1 National Institute for R&D of Isotopic and Molecular Technologies, P.O. Box 700, 400293 Cluj-Napoca, Romania; 2 Service Commun des Laboratoires, 3 avenue du Dr. Albert Schweitzer, 33608 Pessac, France

**Keywords:** Metabolomics, Environmental biotechnology

## Abstract

The potential of excitation - emission matrices (EEM) measurements using classical right angle technique, in conjunction with chemometrics, was prospected for white wine classification with respect to their cultivar and geographical origin. For this purpose, wines belonging to four cultivars (Chardonnay, Pinot Gris, Riesling and Sauvignon) from two different countries (Romania and France) were investigated. The excitation – emission matrices were statistically processed using parallel factor analysis (PARAFAC). According to Soft Independent Modeling Classification Analogy (SIMCA) model, for cultivar differentiation, only 3 out of 107 wine samples (1 Pinot Gris (Romania); 1 Riesling (Romania) and 1 Sauvignon (France)) were misclassified while for geographical origin assessment, only 2 wines (1 Romania and 1 France) were misclassified. This study  demonstrates the potential of excitation – emission fluorescence matrices spectroscopy using the classical right angle technique in wine authentication, without sample dilution.

## Introduction

The development of new reliable analytical methods, able to detect different types of food and beverages frauds, represents nowadays, a constant preoccupation of research and control laboratories. From all investigated matrices, wine represents one of the most studied commodities. Beside the already acknowledged methods for wine control, new analytical approaches (IR, Raman/SERS, ^1^H-NMR, fluorescence spectroscopies) in conjunction with chemometrics, were successfully applied for wine classification^1–5^. One of the analytical techniques which present a high potential for wine cultivar discrimination is represented by the three – dimensional fluorescence spectroscopy, which is a fast, noninvasive, sensitive and affordable technique, involving successive acquisitions of excitation or emission spectra at multiple emission or excitation wavelengths. The advantage of this analytical technique is that all the information regarding the fluorescence characteristics can be entirely acquired by changing excitation and emission wavelengths simultaneously. The resulting emission – excitation data matrix (EEM) provides a total intensity profile of the sample over the range of scanned excitation and emission wavelength.

During the last years, 3-way fluorescence spectroscopy, using front-face technique, combined with the parallel factor analysis (PARAFAC)^6–8^, was successfully applied for the discrimination of different wine types. PARAFAC decomposes the three – dimensional signal into a fixed number of statistical components, with specific contributions, called scores, describing more specifically the variability of all analyzed EEMs^9^. The advantage of PARAFAC over other statistical methods such as principal component analysis (PCA) is the unicity of the decomposition. The wine fluorescence spectra can be further analyzed using principal component analysis (PCA). While the PCA components do not necessarily have a clear physical meaning, they can be  efficiently used to understand and classify the wine data. Once PCA is performed, one can progress further by modelling the data using Soft Independent Modeling of Class Analogy (SIMCA)^10^. After a SIMCA model is obtained for a wine cultivar, geographic region, etc., the distance of a new sample data to the model is used to determine if the wine belongs to that class or not^11^.

The effectiveness application potential of this analytical technique in wine classification stems from the fact that the types and amounts of fluorescence molecules in wines (i.e. polyphenols, vitamins and amino acids)^12,13^ depend on the cultivar and grapes maturity but also on the wine technology.

The main objective of this paper is to prospect the potential of excitation – emission matrix (EEM) fluorescence spectroscopy, in conjunction with Parallel Factor Analysis (PARAFAC), principal component analysis (PCA) and Soft Independent Modeling of Class Analogy (SIMCA) for the discrimination, with respect to cultivar and geographical origin, of a wine set containing samples with very distinct geographical origins coming from Romanian and France. In this regard, a number of 107 white wine samples, from four cultivars (Chardonnay, Pinot Gris, Riesling, Sauvignon Blanc) were considered for this study. Our choice of the methodology is driven by the need to have an easy, affordable and accessible way to discriminate white wines that typical food control labs can employ.

## Materials and Methods

### Sample description

The sample set, involved in this study, was formed by 107 wines, produced in two countries, Romania and France. Thus, a number of 65 wines originating from Romanian (Ro) vineyards, from five consecutive vintages (2012–15; 2013–13; 2014–15; 2015–11 and 2016–11) and belonging to four cultivars Chardonnay (13), Pinot Gris (9), Riesling (20) and Sauvignon Blanc (23) were chosen. For comparison, 42 wine samples from France (Fr), from two cultivars, Chardonnay (16) and Sauvignon Blanc (26) produced in three vintages (2012–14; 2013–17 and 2017–11) were also involved in this study.

### Fluorescence measurements

The photoluminescence measurements were carried out using an ABLE&Jasco V 6500 spectrofluorometer with a xenon lamp of 150 W. 100 μL wine samples were placed in a micro quartz cell (Starna, 3 × 3 mm, vol.: 300 μL) and the spectra were recorded at ambient temperature. The slits of the excitation and emission monochromators were both set at 5 nm. The number of scans was established, after same experimental measurement, at one, in order to avoid sample alteration by UV excitation beam. At the beginning of each day, the blank is measured and its value is valid for subsequent sample measurements. The acquisition speed was set at 500 nm/min, the response at 1 s, and the data pitch at 1 nm as a compromising solution between noise in the spectra and collection time. The excitation and emission wavelengths range were 250–500 nm and 275–600 nm, respectively, with wavelength increments of 5 nm. The landscapes were registered as multiple emission spectra. The wavelength system was calibrated every day by means of the Raman peak to account for a possible wavelength drift of the instrument. The spectrofluorometer used a conventional right – angle optical setup. Total scanning time per sample was approximately 30 min. The measurements were performed within a short period of time (10 days) to minimize the effect of instrumental fluctuation (e. g. lamp intensity) in a similar manner which was previously reported by other authors^5^.

### Chemometrics methods and software

Principal component analysis (PCA) was performed to make a descriptive analysis of the spectral data and Soft Independent Modeling of Class Analogy (SIMCA) was applied to classify this data.

EEM data analysis was performed by using PLS_Toolbox 7.9.5, demo version (Eigenvector Research Inc., Wenatchee, WA) working under Matlab version 7.1.0 (the Mathworks Inc., Natick, MA). Before analysis, EEMs data were corrected for Rayleigh and Raman scattering by using FLUCUT function, included in PLS_Toolbox. In the development of models for wine samples, according to cultivar and geographical origin, a free trial version of SIMCA 13 (Umetrics Suite of Data Analytics Solutions, Umea, Sweden) was used.

#### Parallel factor analysis (PARAFAC)

PARAllel FACtor analysis of the corrected EEM data was performed, in order to extract the relevant information. To model the set of fluorescence data, the EEMs of the 107 samples were arranged in a three-dimensional array ***X*** of size $I\times J\times K=107\times 51\times 66$, where *I* is the number of samples, *J* is the number of emission wavelengths, and *K* is the number of excitation wavelengths. The PARAFAC decomposition can be written as:

1 $${x}_{ijk}=\mathop{\sum }\limits_{f=1}^{F}{a}_{if}{b}_{jf}{c}_{kf}+{e}_{ijk}$$

where F is the number of PARAFAC components. The matrix **A** with matrix elements *a*_*if*_ is called score, while the matrices **B** and **C** are the emission and the excitation loadings, respectively.

In the simplest model, the fluorescence data can be seen as the sum of the signals coming from a number of non-interacting fluorophores. The PARAFAC decomposition is very similar to this sum, and includes all the deviations from this simple model in the array of residuals ***E*** of elements *e*_*ijk*_. The PARAFAC decomposition is made such that the norm of ***E*** is minimal. Within this model, the score matrix element *a*_*if*_ can be interpreted as the concentration of the fluorophores *f* in the sample *i*. The loading matrix element *b*_*if*_ is a scaled estimate of the emission spectrum of the *f*-th fluorophore at the *j*-th frequencey, while the loading matrix element *c*_*kf*_ is proportional to the absorption coefficient of the fluorophore at the *k*-th excitation frequency^5,14^. A non-negativity restriction must be imposed since concentration and emission/excitation coefficients cannot be negative. Percentage of core consistency were calculated in all cases, using the CORe CONsistency DIAgnotic test (CONCORDIA) in order to have an initial idea about the optimal number of components^5^.

#### Principal component analysis (PCA)

The multivariate statistical method used for wines classification are principal component analysis (PCA) and soft independent modeling of class analogy (SIMCA). For both methods standardization is performed by grouping data by each grape cultivar: Chardonnay (C), Pinot Gris (PG), Riesling Italian (R) and Sauvignon Blanc (S) and wine geographical origin: Romania (Ro) and France (Fr).

To perform principal component analysis on fluorescence data, at a fixed excitation frequency, labeled k, we can form a two-dimensional *I* × *J* matrix **X** from the original *I* × *J* × *K* array ***X***. The goal of PCA is to reduce the size of the data matrix X by removing redundant data. To that end, we need to find a new set of axes in the *J*-dimensional frequency space and project the data onto them. The axes need to be chosen as the ones on which the data has maximum variance. It turns out that the unit vectors along this new set of axes are eigenvectors of the matrix **XX**^**T**^. Along these new axes, the data can be decomposed as^15^:

2 $${x}_{ij}=\mathop{\sum }\limits_{r=1}^{R}{t}_{ir}{p}_{jr}^{\text{'}}+{e}_{ij},$$

where *R* is the number of components, or the rank of the decomposition. The *I*-dimensional vectors **t**_*r*_ represent the scores, or the coordinates of the samples in the new principal component space^16^. The loadings *P*_*r*_ represent the proportions, or weights by which the old variables enter into the principal component *r*. The prime symbol in Eq. (2) is for matrix transposition. The idea of PCA is to retain only the only the components that explain most of the variance in the sample data, so *R* is considerably smaller than *J*. The matrix *E* of components *e*_*ij*_ takes care of the errors in representing the data in terms of a few principal components.

#### Soft independent modeling of class analogy (SIMCA)

SIMCA method for pattern recognition and classification was introduced^10^ as a tool for drug design. Subsequent research^17–19^ has improved upon the reliability and robustness of the method. SIMCA relies on PCA for data classification. Typically, from the score plots obtained after performing PCA on the data, one can already see the grouping of the samples. SIMCA is a method to model these groups of data, or classes. The SIMCA models are then used to determine if other samples belong or not to the classes.

For example, to classify wines according to cultivar, one selects a subset of data, called a training set, which contains subsets of wine samples of known cultivar. PCA is then independently performed on each subset of known cultivar. The subset of samples would fill a volume in the subspace spanned by the principal components. The shape of the surface enclosing this volume is sensitive to outliers, so the next step is to remove true outliers. This is done by comparing the distance of the sample to the principal component subspace to a critical distance *s*_0_ specific to the class. Once this is done, a model exists for data corresponding to each wine cultivar. To determine if new data belongs to a given class, its distance to the class model is computed and compared to *s*_0_.

SIMCA is a method to classify data and is built around PCA. For example, if the cultivar is known for some of the wines, one forms a training set containing the fluorescence data for those wines. Each class of the training set contains the wine samples for a given cultivar. One performs PCA on a given class and then uses SIMCA to determine new elements and outliers of that class. SIMCA models are sensitive to fake outliers, i.e. samples belonging to a given class, but very far from other members of that class, and there are various methods to mitigate their effect on the models.

The main difference between PCA and SIMCA is the supervised classification. In the case of PCA classification, a sample is spatially positioned with respect to its analytical data measure for each variable (*i.e*. fluorescence wavelength). This spatial organization of the samples is realized independently of the class of the sample. At some point, samples grouping (clouds) can be observed. These are the PCA classes. SIMCA is classifying the sample according to their class by searching among the samples’ analytical data some discriminant analogies among the classes. Thus, SIMCA is a supervised classification method. The mathematical model is assigning a correlation factor to each variable; as a result, a model can be used to predict to which class will an unknown sample belong. Each group is characterized by a centroid and the class distance within the group is a factor providing the power of the model for class discrimination. The larger this class distance, the better is the model.

Model validation can be achieved in two different ways. The first one is leave-one-out cross-validation (LOOCV). During the model elaboration, intermediate models are built after some samples are excluded. These samples are then classified using this intermediate model. Then, the excluded samples are re-integrated in the sample matrix and another set of samples is excluded. This operation is repeated for all the samples. When there are enough authentic samples, the second possibility is to split them into two batches, one for the model and one to test the model. In both validations, the result is a diagonal-only classification table only if the model discriminates the samples well enough.

In this work, the samples are the wines coming for Romania and France. The variables of the fluorescence data table are the fluorescence absorption and emission wavelengths, while the defined classes differentiate the samples by country and four cultivars (Chardonnay, Pinot Gris, Riesling, Sauvignon Blanc).

## Results and Discussion

### Three - dimensional fluorescence spectra of wines

**Figure 1:**
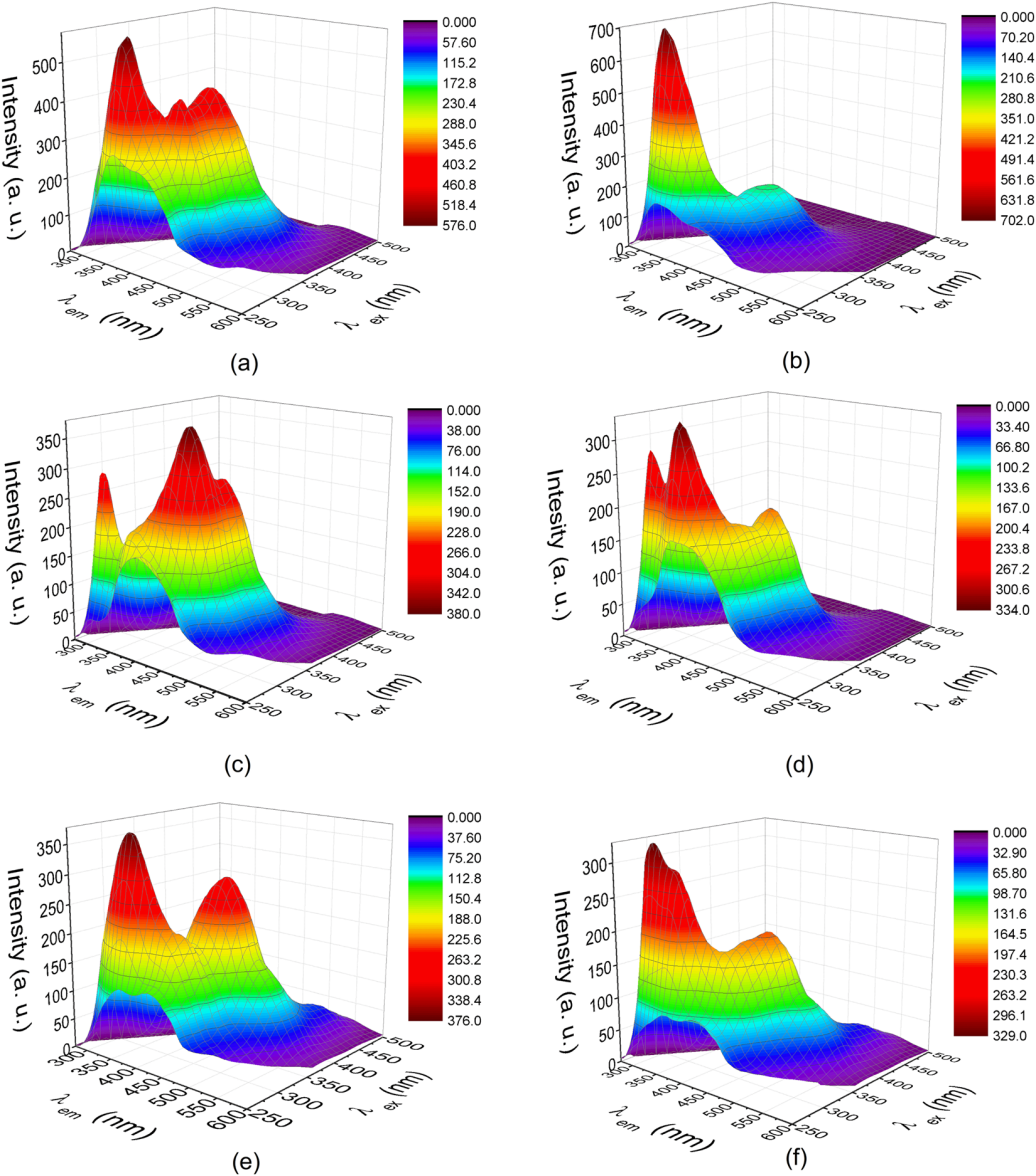
Typical surface excitation – emission matrices for samples of class (**a**) Chardonnay - Ro, (**b**) Pinot Gris - Ro (**c**) Riesling - Ro, (**d**) Sauvignon - Ro, (**e**) Chardonnay - Fr and (**f**) Sauvignon – Fr.

The typical EEM surface of each investigated wine cultivar is presented in Fig. 1. The shape of the EEM, obtained for each cultivar, shows a specific profile containing several fluorophores, which allows the observation of several differentiations, with regard to the wine cultivar (Table 1). Because of the wide range of naturally occurring fluorescent compounds, that exists in a wine sample, the specific emission – excitation matrix represents an overlapped signal of the fluorophores individual contribution.

**Table 1:** Fluorescence spectral characteristics by wine cultivar.

Wine cultivar	Peaks location λ _ex_/λ _em_ (nm)				Fluorescence intensity (a. u.)			
	I	II	III	IV	I	II	III	IV
Chardonnay	280/365	295/415	305/430	355/430	575	390	427	435
Chardonnay - Fr	280/360	295/420	300/420	360/445	375	217	215	300
Pinot Gris	280/325	340/445	—	—	700	215	—	—
Riesling	265/385	280/305	310/435	350/450	207	295	375	285
Sauvignon	280/310 280/370	305/435	355/445	—	285 335	188	200	—
Sauvignon - Fr	280/320 280/360	315/435	340/450	—	328 355	188	200	—

According to the spectra presented in Fig. 1a–d, there are differences in peaks/shoulders numbers among Romanian wine cultivars. Thus, wine cultivars are characterized by different number of peaks, as follows: Chardonnay - four (excitation/emission wavelengths: 280/365 nm, 295/415 nm: 305/430 nm and 355/430 nm); Pinot Gris - two (280/325 nm and 340/445 nm); Riesling - four (265/305 nm, 280/305 nm, 310/435 nm and 350/450 nm) while Sauvignon - four (280/320 nm, 280/360 nm, 305/435 nm and 355/445 nm). Apparently, beside differentiation in terms of peak numbers and general spectra shape, that exists among investigated cultivars, the specific fingerprint of each variety could also be observed based on individual intensities of characteristic signals (Table 1).

A comparison of Romanian wine samples (Chardonnay and Sauvignon), with their corresponding French sorts (Fig. 1a–f), revealed high similarities among their general shapes, suggesting that an evident cultivar fingerprint is present in EEM spectra. Moreover, the fluorescence profile of French wines (Fig. 1e,f), shown four main signals, similar with their corresponding Romanian cultivars (Table 1). Despite this, it could be observed that the peak center of the maxima vary slightly among wines, shifts that will appear due to the natural existing differences among the samples, in terms of their general composition.

Nevertheless, by comparing the EEM wine spectra of samples originated from the two countries, a geographical influence, observed mainly in terms of signals relative intensities could also be intuited, making this technique a possible tool for geographical discrimination, as well.

### Results of the chemometric approaches for cultivar differentiation

To classify wines by their cultivar, we have used three chemometric methods, parallel factor analysis (PARAFAC), principal component analysis (PCA) and SIMCA which builds its data models on the PCA results.

PARAFAC models were built in order to extract the characteristic excitation and emission profile of the main fluorophores characteristic to each wine cultivar. The optimum number of factors for each PARAFAC model was selected comparing the quality parameters of the model built for an increasing number of factors and the best models obtained were 2-factor PARAFAC models.

**Figure 2:**
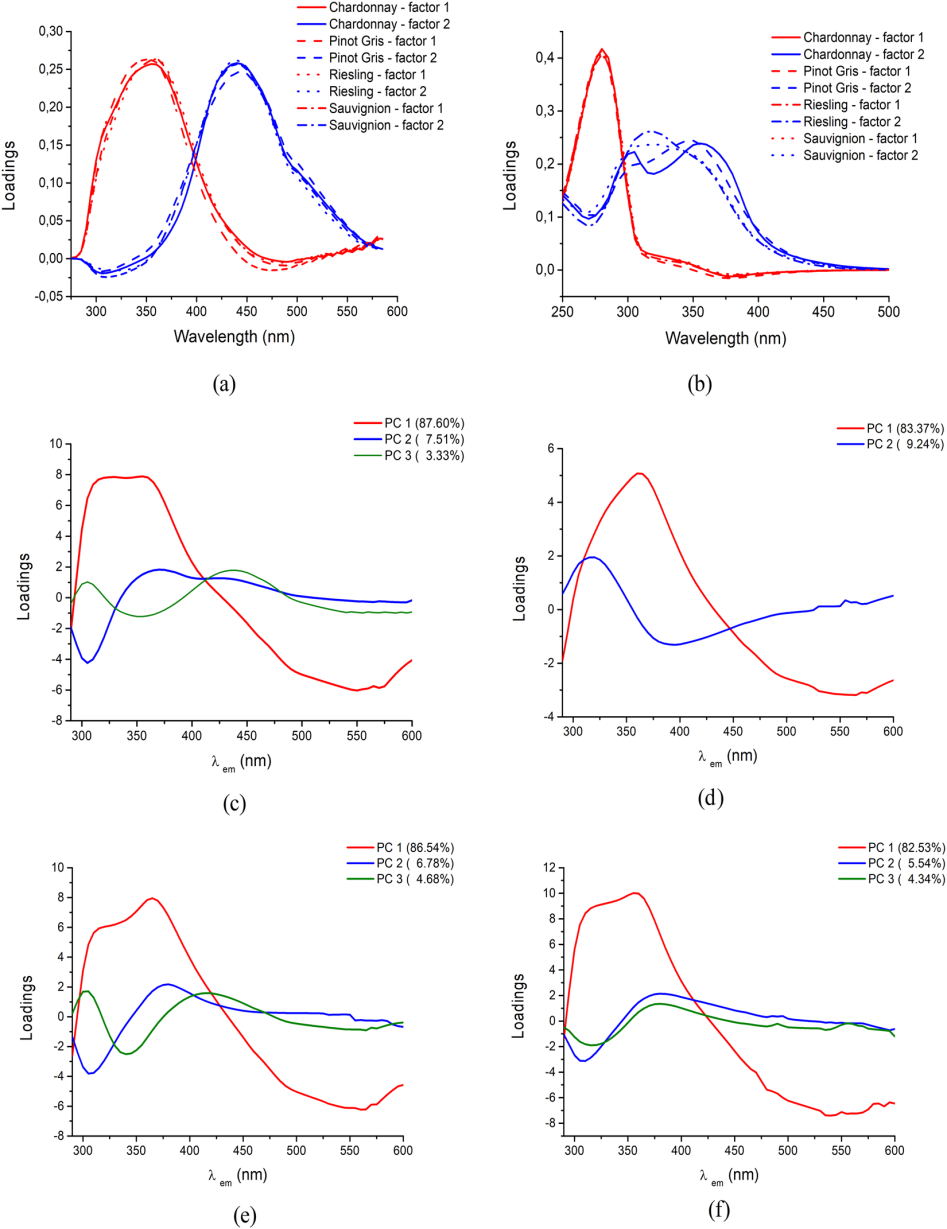
PARAFAC fluorescent loadings for the two components of the non-negativity constrained PARAFAC model constructed on the basis of the fluorescent EEM of 107 wine samples and results of principal component analysis carried out with λex = 280 nm. The loadings of the first principal components (PC1 – PC2 or PC1 – PC3) as well as the corresponding explained variance (%) are shown. (**a**) excitation, (**b**) emission. factor 1 in red, factor 2 in blue. (**c**) Chardonnay, (**d**) Pinot Gris, (**e**) Riesling and (**f**) Sauvignon.

Factor 1 (red in Fig. 2a,b) has a maximum excitation at 280 nm for maximum emission at 350–360 nm. As previously reported in literature, the peaks in this region are characteristic to tryptofan, gallic and protocatechuic acids^13,20–22^. The pair of excitation/emission wavelengths corresponding to the maximum fluorescent intensity for the second component (blue in Fig. 2a,b) is 304–350/430–440 nm. According to the literature, the excitation/emission wavelengths of this factor are characteristic to phenolic acids^23^ and phenolic aldehydes^24–26^. Phenolic compounds represent the best-known fluorescent molecules that are naturally present in wine and which are directly related to grape cultivar and wine aging^26^. The small shifts that appear in the characteristic excitation/emission pairs of a certain wine compounds, from one sample to another, are due to the different vicinities of molecules, that conduct to a slightly different environment^5^.

PCA was carried on the emission spectra to investigate the samples grouping with respect to wine cultivar. A good classification using CORCONDIA was achieved using the first two or three PCs of the PCA performed on the emission spectra recorded at the excitation wavelengths at λ_ex_ = 280 nm, which represent the best predictor for the phenolic contents in wine^27^ and emission spectra between 295–600 nm. For the fluorescence emission data collection, discrimination as function of wine cultivar was achieved.

Chardonnay cultivar (Fig. 2c) is characterized by three fluorescent components PC1, PC2 and PC3 which, explains the variability as follows: PC1 = 87,6%, PC2 = 7,51% and PC3 = 3,33%. First component PC1, comprised two main positive loadings located at 315 nm and 360 nm, respectively. The loading from 315 nm is characteristic, according to previously reported studies, to flavonoid class (catechin and epicatechin)^28^ while, the second loading from 360 nm, could represent overlapped signals coming from different wine constituents like: phenolic acids: gallic^24^, syringic acid^25^, phenolic aldehydes (2,5 Dihydroxybenzaldehyde)^25^ and amino acids (tryptofan)^20^. PC2 is characterized by positive loadings at: 433 nm, and around 361 nm, attributed to overlapped signals of catechin and epitatechin^25^, gallic^24^ and protocatechuic^25^. Apart from this, were observed negative loadings for a band centered at 305 nm, representative for tyrosine^7^, indicating a negative relationship between these different classes of fluorescence molecules present in wines.

The last principal component PC3 is formed by two main loadings signals centered at 305 and 436nm^29^, which are typical bands for tyrosine and tyrosol^30^ and phenolic compounds of the type of chlorogenic acid, caffeic acid, coumarins and stilbenes, respectively^31,32^.

Pinot Gris cultivar (Fig. 2d) is defined by two components PC1 (83.37%) and PC2 (9.24%). The first component (PC1) profile has a narrow maximum centered around 327 nm due to p-hydroxybenzoicacid^33^ and also a sharp emission maximum at 363 nm. The pair of excitation/emission wavelengths corresponding to the fluorescence intensity for PC2 is 280/317, associated with catechin and epicatechin^28,33^. For this second component a negative loading centered at 396 nm, typical for naturally fluorescent compounds present in wine, was obtained^24^.

Riesling cultivar (Fig. 2e) is characterized by three principal components that are defining its specific fingerprint in distinct total variance: PC1 (86.54%), PC2 (3.78%) and PC3 (4.68%). The 280/315 wavelength, characteristic for flavonoid class (catechin and epicatechin^28^) and 280/365 nm which might come for overlapped signals from different phenolic acids^25^, represents the PC1. The pair excitation/emission maxima at 280/376 nm from the second component, PC2, originates from phenolic compounds such as gallic^24^ and/or syringic acid^25^. Finally, PC3 (comprises two components the one from 280/303 nm, attributed to amino-acids (tyrosine and tyrosol)^30^, and the loading from 280/411 nm which could appear due to the presence of fluorescent cinnamic acids^7^.

For Sauvignon Blanc cultivar, the first component PC1 accounted for a percentage of 82.53% to its characteristic pattern (Fig. 2f). This component has two pairs of excitation/emission wavelengths at 280/313 nm and 280/358 nm being characteristic to flavonoid class (catechin and epicatechin)^25,28^. The spectral pattern associated with the PC2 and PC3 exhibited positive loadings at 380 nm, matches with stilbenes compounds such as trans-resveratrol^34^ and negative one at 303, characteristic for tyrosine^7^.

The obtained results after PCA statistical treatment, suggested the possibility to classify the sample set based on wine cultivar. Based on this assumption, SIMCA classification was performed, using three - dimensional fluorescence spectra of the entire wine set. SIMCA enables the sample classification into an already existing group, assigning new objects to the class to which they show the largest similarity.

For the cultivar discrimination of wine samples, the PCA classes, previously established for each wine sort: Chardonnay (C), Pinot Gris (PG), Riesling (R) and Sauvignion Blanc (SB) were used in the development of SIMCA model.

**Figure 3:**
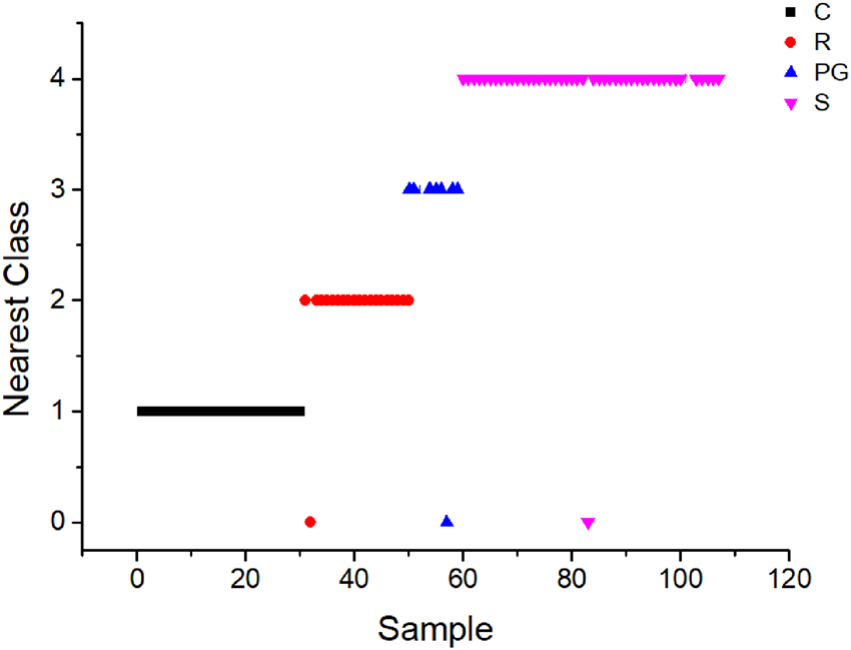
Nearest classes for SIMCA submodels.

The achieved classifications, according to SIMCA model are shown in Fig. 3 and Table 2 (Supplementary). According to the SIMCA models, only 3 out of 107 Romanian and French wines were misclassified at a 95% confidence level. The misclassified samples belonged to both countries, as follows: 1 Pinot Gris (Romania); 1 Riesling (Romania) and 1 Sauvignon (France). A R-Square value of 0.985 was obtained for this this model while its capacity of prediction was situated around 98%.

By default, SIMCA 13 uses a leave-one-out cross validation method. In one round of cross validation, row data (observations) is left out of the model, then loading vectors without this data are calculated. Then column data (variables) is left out and scores are calculated. The data that was left out, is then predicted from the model. If the data predicted from the model is close enough to the original data, then the model is valid. These steps are performed for each row and column of the data, and at each of these cross-validation rounds, a prediction error is calculated by summing the squares of the differences between the original and predicted row (column) elements.

### Classification of wines with respect to geographical origin

**Figure 4:**
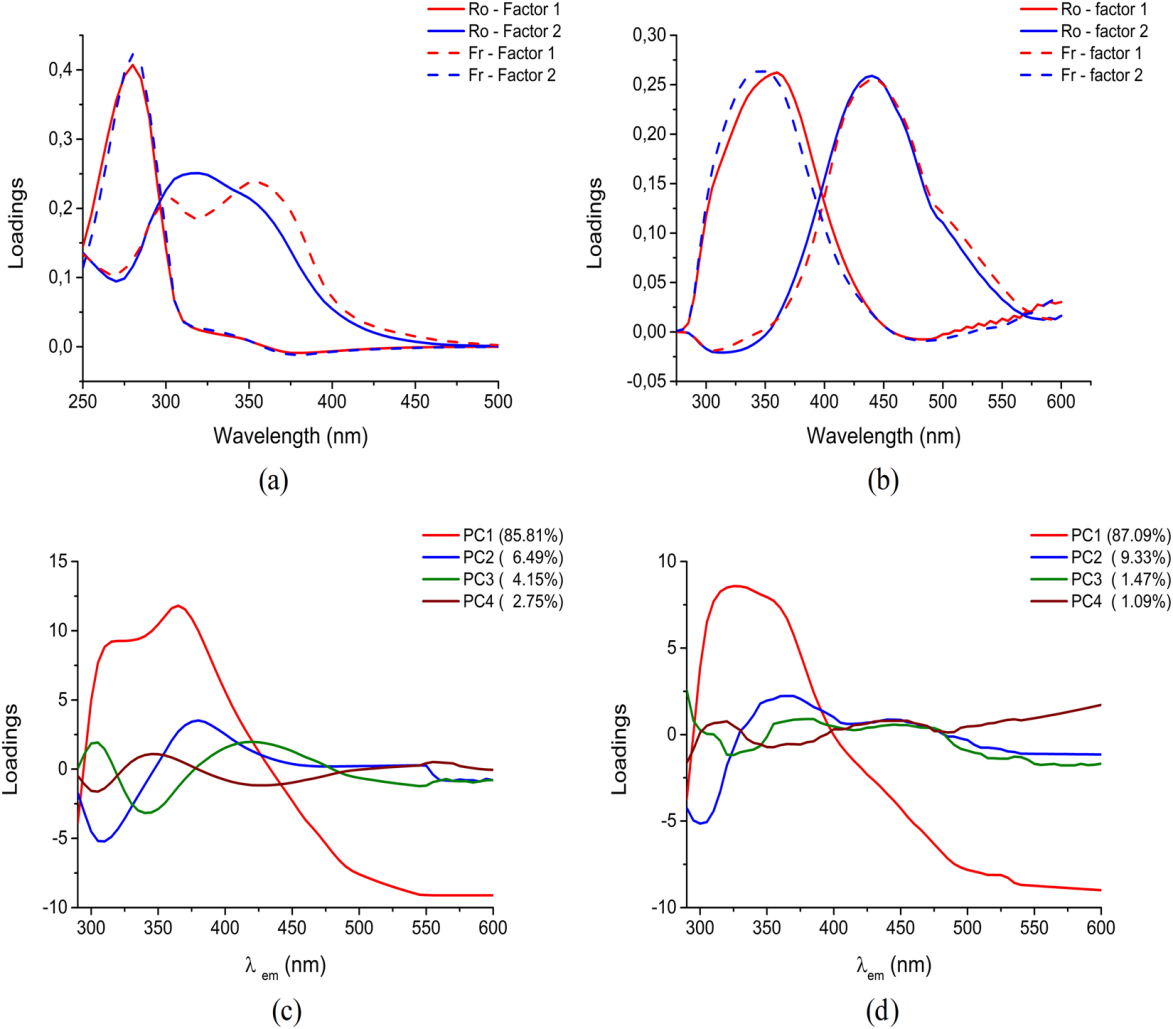
PARAFAC fluorescent loadings for the four components of non-negativity contained PARAFAC model constructed on the basis of the fluorescent EEM and results of principal component analysis carried out with λ_ex_ = 280 nm. (**a**) excitation. (**b**) emission, factor 1 in red, factor 2 in blue, (**c**) Romanian and (**d**) French wine.

For the geographical differentiation of samples (Romania vs. France) it was obtained that the optimum number of factors, for each PARAFAC model (Fig. 4a,b) is 4.

After performing PCA, the best classification was achieved using emission spectra (290–600 nm) recorded at excitation wavelength 280 nm. The fluorescence spectra showed different shapes, given mainly by the distinct bands intensities of each individual compounds as well as, small signals shifts due to the particular compositions of wine samples.

Romanian wines (Fig. 4c) are characterized by four principal components PC1, PC2, PC3 and PC4, that are defining their specific fingerprint in a distinct variance (PC1 = 85.81%, PC2 = 6.49%, PC3 = 4.15% and PC4 = 2.75%). First component PC1 is formed by two main positive loadings signals centered at 310 nm and 365 nm which are typical bands for catechin, epicatechin^25,34^ and gallic acid^24^. PC2 is characterized by a negative loading at 305 nm, which is a typical signal of tyrosine and tyrosol^7^ and also by a positive loading at 380 nm, representative for stilbenes compounds such as trans-resveratrol^34^. PC3 is characterized by positive loadings at: 303 nm, a typical signal of tyrosine and tyrosol^30^, 420 nm attributed to fluorescent cinnamic acids^7^, and as negative loading, a band centered at 340 nm.The last principal component PC4 is formed by negative loadings at 303 nm associated with tyrosine and tyrosol^30^, and another one around 430 nm. Apart of these, another pair 280/345 nm, responsible for amino acids was obtained.

French wines (Fig. 4d) are categorized by four fluorescent components PC1, PC2, PC3 and PC4, that are defining their specific fingerprint in distinct total variance: PC1 = 87.09%, PC2 = 9.33%, PC3 = 1.47% and PC4 = 1.09%.

The first component (PC1) is a broad peak centered at 325 nm with a shoulder at 353 nm, which is due to overlapped signals of p-hydroxybenzoic acid^33^, catechin, epicatechin^33^. The profile loadings for PC2 shown a negative loading at 300 nm matching with tyrosine and tyrosol^7^, and two positives at 360 nm which agrees with the presence of gallic^24^, syringic acid^25^, and a broad emission from 400–500 nm. The later loading matched with individual molecules, such as: gentisic acid, flavonols (quercetin, quercitrin), vitamins i.e. riboflavin, or more condensed structures involving quinone moieties^12,13,23,29,35^.

The component PC3 matched well with the catechin and epicatechin (310 nm)^34^, anthocyanins (370 nm) and gentisic acid, flavonols like quercetin, quercitrin, vitamins, quinone (445 nm)^12,13,23,29,35^.

The fourth principal component analysis shows a negative loading at 300 nm typical signal of tyrosine and tyrosol^30^ and two positive loadings at 360 and 445 nm.

**Figure 5:**
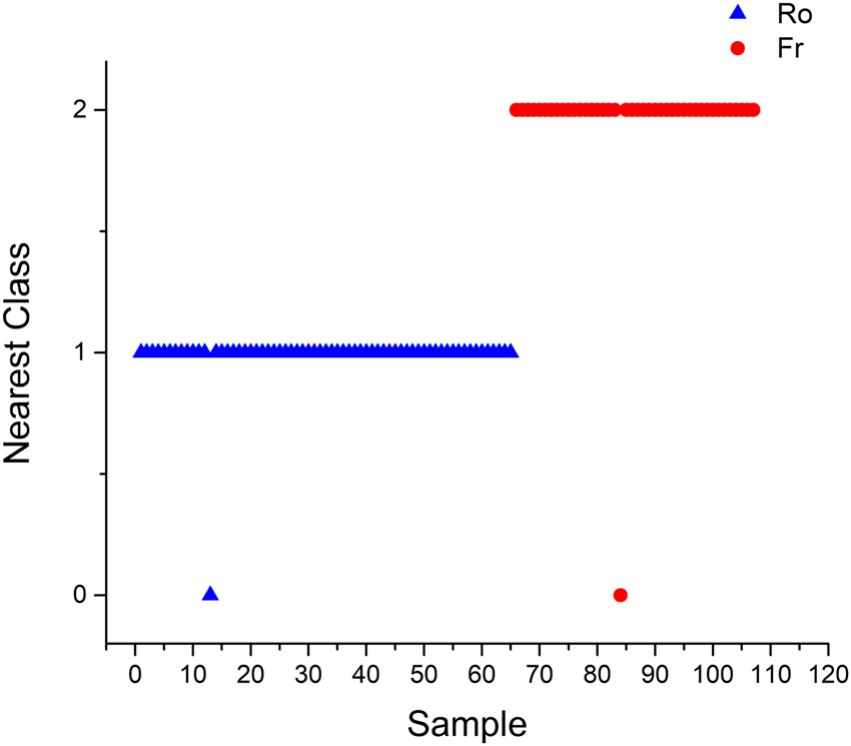
Nearest classes for SIMCA submodels.

SIMCA models were developed using previously established PCA classes for each wine sort: Romanian (Ro) and France (Fr). According to the SIMCA models (Fig. 5 and Table 3-Supplementary), only 2 out of 107 Romanian and France wines were misclassified at a 99% confidence level. The 2 misclassified samples belonged to both countries: 1 Romania and 1 France. For this model the R-Square value was 0.925 and the capacity of prediction around 96%.

## Conclusion

A data set of 107 wines belonging to four cultivars (Chardonnay, Pinot Gris, Riesling and Sauvignon) from two different countries (Romania and France) was evaluated by applying excitation – emission fluorescence matrices spectroscopy in conjunction with chemometrics, in order to classify the samples with respect to their cultivar and geographical origin.

A simple visual characterization of EEMs typical surface, pointed out specific profiles, containing several fluorophores, characteristic to each cultivar, with peak maxima that slightly vary among wines. Moreover, the comparison of Romanian wine samples (Chardonnay and Sauvignon), with their corresponding French sorts indicated the presence of a cultivar fingerprint in EEM spectra. The geographical influence, observed in EEM wine spectra, is mainly emphasized through differences in relative intensities of the characteristic signals.

PARAFAC gave information about the potential fluorescent compounds present in each wine, allowing their differentiation according to their cultivar and geographical origin. A good classification was achieved using the first three PCs of the PCA for the cultivar and the four PCs of the PCA for the geographical discrimination, performed on the emission spectra recorded at the excitation wavelengths at λ_ex_ = 280 nm. The developed SIMCA models proved a higher capacity of prediction: 98% for the simultaneous cultivar classification and 96% for the geographical discrimination.

## Supplementary information

Tables 2 and 3
