# Supplementary material for: Application of fluorescence spectroscopy using classical right angle technique in white wines classification
Source: Sci Rep. 2019 Dec 3;9:18250. doi: 10.1038/s41598-019-54697-8 (PMC6890751; doi:10.1038/s41598-019-54697-8)
Supplement: Supplementary file 1 — Tables 2 and 3 [file 41598_2019_54697_MOESM1_ESM.pdf]

Application of fluorescence spectroscopy using classical right angle technique in white wines classification

Ramona-Crina Suciu[1], Liviu Zarbo[1], Francois Guyon[2], Dana Alina Magdas[1*]

[1]*National Institute for R&D of Isotopic and Molecular Technologies, P.O. Box 700, 400293 Cluj-Napoca, Romania*

[2] *Service Commun des Laboratoires, 3 avenue du Dr. Albert Schweitzer, 33608 Pessac, France*

*Corresponding author: Dana Alina Magdas

E-mail address: amagdas@itim-cj.ro

Table 2. Results of modeling analysis of SIMCA (Cultivar differentiation)

Actual	# of PC	Predicted class			
		Class C	Class R	Class PG	Class S
Class C (30) *	3	30	0	0	0
Class R (20) *	3	0	19	0	0
Class PG (9) *	2	0	0	8	0
Class S (48) *	3	0	0	0	47
* number of samples per class					

Table 3. Results of modeling analysis of SIMCA (Geographical differentiation)

Actual	# of PC	Predicted class	
		Class Ro	Class Fr
Class Ro (65) *	4	64	0
Class FR (42) *	4	0	41
* number of samples per class			
